# Ultrasonic Non-Destructive Testing System of Semi-Enclosed Workpiece with Dual-Robot Testing System

**DOI:** 10.3390/s19153359

**Published:** 2019-07-31

**Authors:** Canzhi Guo, Chunguang Xu, Juan Hao, Dingguo Xiao, Wanxin Yang

**Affiliations:** 1 School of Mechanical Engineering, Beijing Institute of Technology, Beijing 100081, China; 2 Fundamental Science on Advanced Machining Laboratory, Beijing 100081, China

**Keywords:** ultrasonic testing, robotic non-destructive testing, trajectory planning, dual-robot system

## Abstract

With the rapid development of material science, more and more workpieces, especially workpieces with complex curved surfaces, are being made of composite materials. Robotic non-destructive testing (NDT) systems for complex curved surface composite material parts are being used more and more. Despite the emergence of such flexible NDT systems, the detection of semi-enclosed parts is also a challenge for robotic NDT systems. In order to overcome the problem, this paper establishes an NDT solution for semi-enclosed workpieces based on a dual-robot system of synchronous motion, in which an extension arm is installed on one of the robots and presents a trajectory planning method that always ensures the extension arm is parallel to the rotary axis of a semi-enclosed workpiece and that the ultrasonic probes are perpendicular to the workpiece surface. Trajectory analysis experiments and ultrasonic NDT experiments utilizing the optimal water path distance determined by simulation result of multi-Gaussian beam model for two types of semi-enclosed workpieces are performed with the dual-robot NDT system. Experimental results prove that the dual-robot NDT scheme functions well and the planned trajectories are correct. All the hole-shaped artificial defects with diameters ≥3 mm are detected by using 2.25 MHz ultrasonic probes through the transmission testing method. Vivid 3D C-scan image of a small diameter cylindrical workpiece based on the testing result is provided for convenience of observation.

## 1. Introduction

It is well-known that composite materials are used in various industries, especially in aviation and aerospace industries, due to the desire to avoid corrosion, to enhance impact and fatigue resistance, and to develop lighter structures [1]. The qualities of composite components are critical to life safety, so the integrity of these components has become a core problem that engineers are very concerned about. Non-destructive testing (NDT) techniques are used to inspect internal and surface defects such as cracks, voids, porosity, inclusions, and corrosion [2]. Previously, the time-consuming, laborious manual testing method has been commonly used for these workpieces, which has provided motivation to develop automated NDT equipment for these components. Some NDT techniques, such as ultrasonic testing (UT), eddy currents testing (ECT), and radiography testing (RT), can be readily automated by the use of wheeled, climbing, crawling, or joint-type industrial robots [2].

In recent years, research regarding robotic NDT technology and its relative equipment has been very active. Sattar and Brenner proposed a position-force-moment (PFM) feedback method which adapted a seven-axis robot arm to scan a complex-shaped part by keeping the NDT probe perpendicular to the surface and maintaining a constant contact force with the part surfaces [3]. However, in order to maintain high reliability, the system must reduce the detection speed. Xiao [4,5] established an NDT system in which the probe was fixed and the workpiece was grasped by the robot for movement. This system is well-suited for the high-speed automatic testing of small complex-shaped parts, such as aircraft engine blades. From his research results, he also showed a method to calibrate the end effector orientation using the ultrasonic alignment method, in order for the robot to adjust the orientation of the tool frame to keep the probe perpendicular to the surface. Further, for large curved rotators, such as wheel hubs, Zhang [6] developed another robot-assisted UT system and in order to improve the inspection sensitivity, the focus depth of the ultrasonic beam was optimized.

Although the robotic NDT method has many merits compared with those of other NDT methods, sometimes a system with only a single industrial robot is not sufficient to inspect complex curved composite parts with s high ultrasonic attenuation rate, such as carbon fiber materials. In this case, the importance of the dual-robot NDT system is highlighted. Maurer concluded that the dual-robot automatic scanning system became increasingly used to inspect carbon fiber parts, such as aircraft fuselages [7]. For multi-robot systems, the relationships between various coordinate frames are very important. Lu presented a simple “THREE POINTS” method for synchronizing the part frames of a dual-robot cell in NDT systems [8]. Cooper [9] and Mineo [1] established a twin-robot NDT system with a simple-to-use graphical user interface (GUI) to control all aspects of the automatic inspection, from the trajectory data loading to the scanning data analysis. The system could also control the robot to return to the points of interest (the points are probably defects), which greatly increased the practical usefulness of the inspection system. In order to perform non-destructive testing of complex curved workpieces, Mineo developed flexible trajectory planning software for manipulators in NDT systems based on the MATLAB toolbox [10]. He also designed a customized instrument, referred to as FIToolbox, to enable high speed analogue acquisition [11]. This was a major advancement in the area of robotic ultrasonic NDT. For the inspection of workpieces with variable thicknesses, Lu [12] proposed a kinematic constraint method between two robots; this method guaranteed that the transmitting transducer was in the normal direction of the incident surface and the receiving transducer was in the sound beam propagation path. Morozov [13] introduced a practical off-line trajectory planning method based on reverse engineering for an unknown model. Guo [14] established a dual-robotic NDT system and presented a four-posture calibration method to calibrate irregular-shaped tools used for complex workpiece testing. Simultaneously, he also proposed another method for aligning the two probes completely during the inspection of the dual-robotic NDT system, which was called the tangential constraint method [15]. In the aspect of multi-axis motion control, Rodriguez-Angeles [16], Sun [17], and Shang [18] proposed a synchronizing controller, a model-free cross-coupled controller, and a task-space coordination controller, respectively, for multi-axis motions. They showed good performance in reducing synchronization errors.

Despite these abundant and accessible documents, the composition and workflow of the dual robot system and the detailed NDT methods for semi-enclosed workpieces have not been described. The crucial challenges include the generation of usable trajectories for complex curved workpiece surfaces, real-time compensation of movement trajectory, and fast data processing. This paper establishes a dual-robot NDT system (DRNDT) for the inspection of semi-enclosed workpieces and presents a specific trajectory planning approach for the NDT scheme to inspect these workpieces. The paper is structured as follows. Section 2 introduces the NDT method of the semi-enclosed workpieces and the composition and working principle of the DRNDT system. A custom trajectory planning method for semi-enclosed workpieces inspection is introduced in Section 3. Verification experiments of the trajectory planning method and ultrasonic testing experiments of a rectangular semi-enclosed box and a cylindrical workpiece are presented in Section 4 and Section 5. The conclusions are provided in Section 6.

## 2. Scheme of the DRNDT System

### 2.1. Architecture of the Dual-Robot NDT System

**Figure 1:**
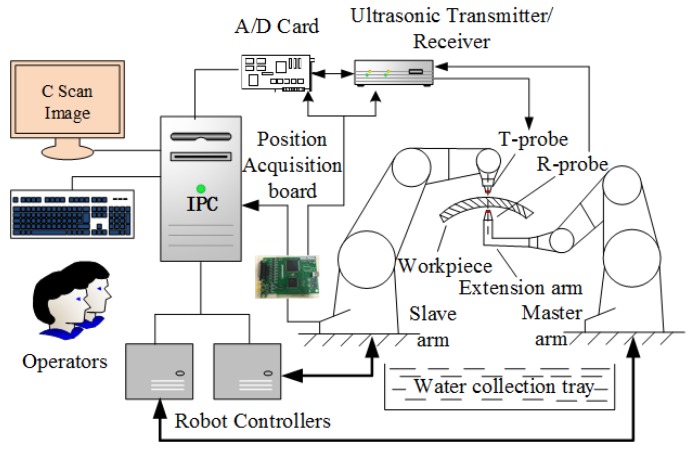
Schematic of the dual-robot non-destructive testing (NDT) system.

The DRNDT system is characterized by multiple degrees of freedom, good flexibility, and a wide range of applications. The architecture of the system is shown in Figure 1. One of the robots held an extended irregular-shaped arm to move in the interior space of a semi-enclosed workpiece for NDT.

**Figure 2:**
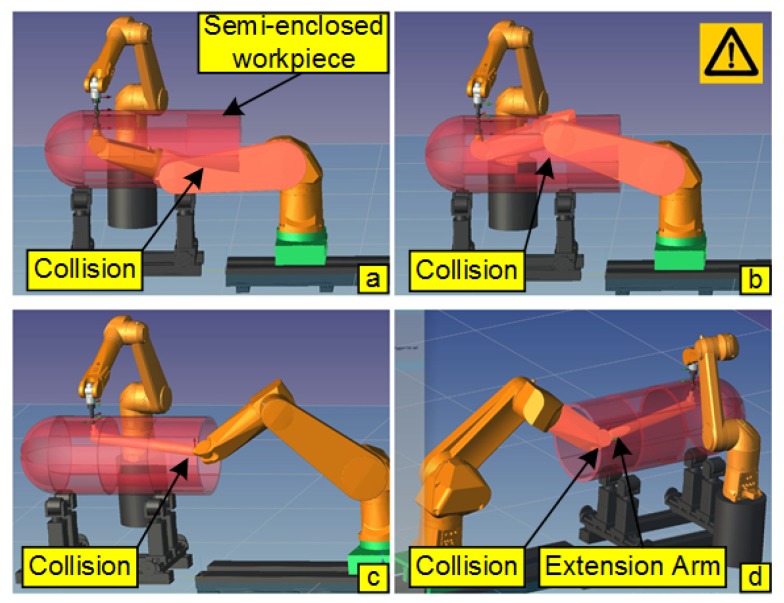
Typical collisions of the dual-robot NDT (DRNDT) system.

This may be the best way to inspect this type of parts. The extension arm was fitted with appropriate constraints to prevent the risk of collision. Typical collision accidents of the DRNDT system with or without an extension arm are shown in Figure 2.

Figure 2a,b show the collisions between the robot and the semi-enclosed workpiece when an extension arm was not deployed on the robot. Figure 2c,d show the collisions between the robot (or extension arm) and the semi-enclosed workpiece when an extension arm was mounted on the robot. In order to ensure that the system performs NDT of the semi-enclosed workpiece without collision, a coordinate frame constraint method and corresponding special post-processing method of trajectory were proposed. Details of the method are explained in Section 3.

**Figure 3:**
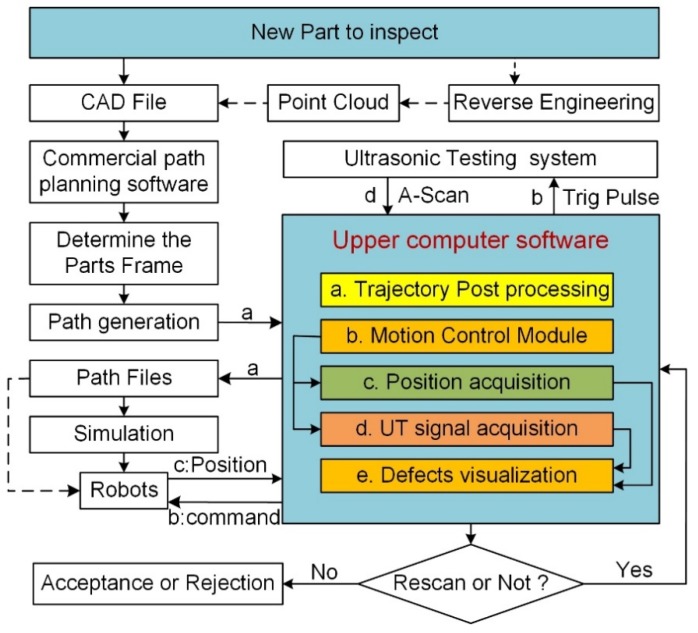
Schematic diagram of the dual-robot NDT procedure. CAD (Computer Aided Design); UT (Ultrasonic Testing).

For easier control of the entire system, a well-functioning upper computer software was also developed for this system. Figure 3 provides the schematic diagram of the overall inspection process, including the interactions between the software and each module. This matching software coded with C# programming language was developed, which integrated all the modules that were needed during the testing process, such as trajectory post processing (module a), motion control (module b), position and ultrasonic data collection (module c and d), and results visualization (C-scan image, module e).

The main operation steps of this system were as follows. First, the 3D model of a workpiece through CAD (Computer Aided Design) files or reverse engineering was obtained. Second, the initial trajectory through commercial CAM (Computer Aided Manufacturing) software such as Dassault DelmiaV5 R20 was planned. Third, post-processing of the trajectory through the upper computer software was performed and the final trajectory files were generated (process “a” of the software in the scheme of Figure 3). Fourth, motion simulation was performed and the trajectory data files were downloaded to the robot controller if there was no risk of collision. Finally, the real machine experiment began and all inspecting processes were controlled by the upper computer software, including the starting and stopping of the motion system and the ultrasonic system simultaneously, ultrasonic data and position data collection synchronously (processes “b”, “c”, and “d”), and defect visualization. In the testing process, once the robot motion was activated, the acquisition module collected position data and the corresponding ultrasonic data at every trajectory increment. Once the data was collected, it was sent to the upper computer software for processing, analysis, and C-scan image display. The realization of the position-driven data acquisition was based on a high-speed robot position acquisition board developed in our laboratory.

This board guarantees a high-speed acquisition of position data and breaks the limitation of the low acquisition rate due to the long system cycles of the robot controller (STAUBLI, a robot brand, robot’s system cycle is often 4 ms). Its highest position acquisition rate can reach 10 kHz and it can obtain position data with an interval of 0.1 mm. When the robot moves, the acquisition board sends position data at a specified trajectory increment and a trigger pulse to trigger ultrasonic data acquisition. This ensures that the position data and ultrasound data are collected at the same time. These well-synchronized position and ultrasonic data further produce good quality 2D and 3D C-scan images.

### 2.2. Coordinate Frames in the Dual-Robot NDT System

**Figure 4:**
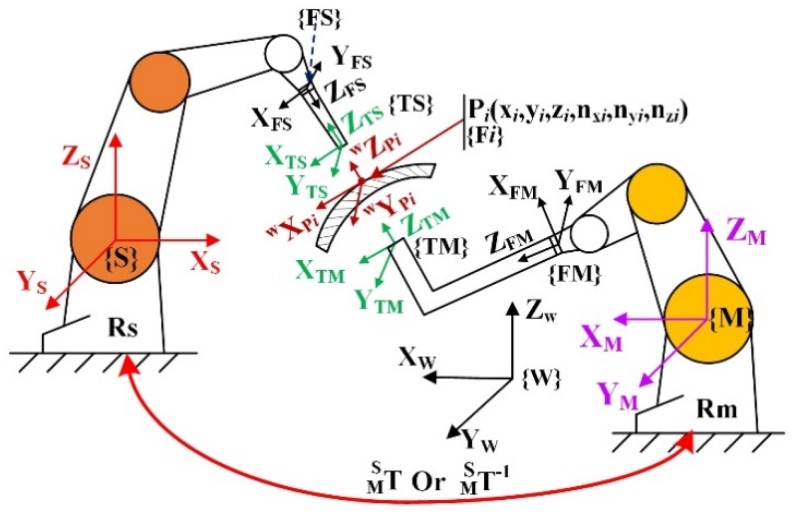
Cartesian frames in the DRNDT system. Rm and Rs are the two robots, master and slave robot. {M} and {S} are the base frames of them. {FM} and {FS} are the flange frames of them. {TS} and {TM} are the tool frames of the two robots. {W} is the workpiece frame.

The key to synchronizing the motion of two robots is that any point in space is mapped into the two robot base frames. Therefore, the definition of the coordinate frames in the DRNDT system should be explained in advance. As shown in Figure 4. Frame {$F_{i}$} is the frame of the discrete point $P_{i}$ on the planning trajectory, called the auxiliary discrete point frame. The expressions of $P_{i}$ in frames {M} and {S} are PiM=[XiM,YiM,ZiM]′ and PiS=[XiS,YiS,ZiS]′. TM S  is the rotation matrix that implies the transformation relationship of frame {M} relative to frame {S}.

To coordinate the movement of the two robots, the coordinates of the testing points on the surface of the workpiece and their normal directions should be known for each robot’s base frame (i.e., {M} and {S}). Usually, coordinate transformations from workpiece frame to robotic base frames are needed, because the discrete points on the trajectory are expressed in frame {W}, rather than expressed in the robotic base frames. If TM S  is known, we can map any point relative to one robotic base frame into the other robotic base frame according to the coordinate transformation theory. In addition, it should be noted that the frame rotation sequence of the robot used in this paper follows the X-Y-Z Euler angle setting convention.

### 2.3. Method to Calibrate the Transformation Relationship of Frame {**M**} and Frame {**S**}

In robotics, a vector is often used to describe coordinate and orientation. It is assumed that there are four non-coplanar points in the communal workspace of the two robots. Their descriptions in frame {M} are PiM=[XiM,YiM,ZiM]′ and in frame {S} are PiS=[XiS,YiS,ZiS]′ (i=1–4). According to coordinate conversion theory, the translation equation is

(1) [PiS1]=TMS⋅[PiM1],

where i = 1,2,3,4.

Expanding Equation (1), yield

(2) [X1SX2SX3SX4SY1SX2SX3SX4SZ1SX2SX3SX4S1111]=TM S ⋅[X1MX2MX3MX4MY1MX2MX3MX4MZ1MX2MX3MX4M1111].

If the four points, $P_{1}$, $P_{2}$, $P_{3}$, and $P_{4}$, are selected in such way that the vectors formed by any two points of them are linearly independent, in other words, |M|≠0, Equation (2) has a unique solution, i.e.,

(3) TM S  =[X1SX2SX3SX4SY1SX2SX3SX4SZ1SX2SX3SX4S1111]⋅[X1MX2MX3MX4MY1MX2MX3MX4MZM1XM2XM3XM41111]−1.

The translation matrix can be determined experimentally. The robots holding the measuring tip move to the four selected points respectively to record the coordinates PiM and PiS of the four points. Then, the matrix TM S  between the two robots can be determined with Equation (3). More profoundly, TM S  is composed of a rotation matrix (RM S ) and a translation vector (PMOriS). PMOriS is the representation of the base point of frame {M} relative to frame {S}.

(4) TM S  =[RMSPMOriS01]

If the rotation matrix RMS does not meet the constraint of orthogonal normalization, it can be normalized orthogonally using the matrix orthonormalization method presented by Lu [8] to normalize the rotation matrix.

### 2.4. Method to Calibrate the Transformation Relationships of a Workpiece Frame Relative to Robotic Base Frames

The transformation relationships between a workpiece frame {W} and robotic base frames {$B_{M}$}/{$B_{S}$} are the basis of the simultaneous movement of the two robots and other robotic work cells. The relationships between them can be determined by calibrating three points on the part using the robots holding measuring tips. These three points are determined as follows:

(1) Point $O_{w}$ ($x_{ori},y_{ori},z_{ori})$ determines the base point of frame {W};
(2) Point $X_{w}$ ($x_{X_{w}},y_{X_{w}},z_{X_{w}}$) determines the X-axis of the frame {W} and the X-axis direction;
(3) Point $Y_{w}^{\prime }$ ($x_{Y_{w}},y_{Y_{w}},z_{Y_{w}}$) determines the XOY plane of the frame {W}.

**Figure 5:**
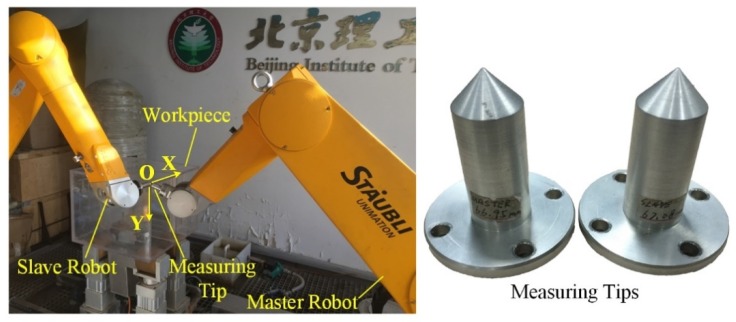
A typical calibration process of workpiece frame.

These three points are denoted as Pmo {$x_{mo},\;y_{mo},\;z_{mo}$}, Pmx {$x_{mx},\;y_{mx},\;z_{mx}$}, and Pmy {$x_{my},\;y_{my},\;z_{my}$} relative to frame {M} and Pso {$x_{so},\;y_{so},\;z_{so}$}, Psx {$x_{sx},\;y_{sx},\;z_{sx}$}, and Psy {$x_{sy},y_{sy},\;z_{sy}$} relative to frame {S}. A typical calibration process of a workpiece frame is shown in Figure 5.

The transformation matrixes between frame {W} and the robotic base frames {$B_{M}$}/{$B_{S}$} can be obtained through the following specific calculation.

The unit vector XWM,YWM,ZWM, of *x*, *y*, *z* components of frame {W} relative to frame {M} can be expressed with the coordinates of the three selected points relative to frame {M}. The calculation method is as follows.

(5) {XWM=(xmx−xmo, ymx−yyo, zmx−zmo)(xmx−xmo)2+(ymx−yyo)2+(zmx−zmo)2Yw′M=(xmy−xmo, ymy−ymo, zmy−zmo)(xmy−xmo)2+(ymy−ymo)2+(zmy−zmo)2ZWM=XWM×Yw′MYWM=ZWM×XWM,

where Yw′M is an arbitrary vector on the XOY plane point to the positive direction of Y-axis of frame {W}. 

The transformation relationships of frame {W} relative to frame {M}/{S} are shown in Equations (6) and (7), respectively.

(6) TwM =[XWTMYWTMZWTMPmo0001]

(7) TwS =[XWTSYWTSZWTSPso0001]

Another method to determine the relationships between a workpiece frame and the robotic base frames are to first determine the relationship relative to one robot by using one of the robots to calibrate the three points of the workpiece frame, then determine the relationship of the workpiece frame relative to another robotic base frame according to Equation (4). According to the matrix transformation theory, the equations for calculating the relationships of frame {W} relative to frame {M}/{S} by this method are as follows.

(8) TwS =TM S ⋅TwM

(9) TwM =T−1M S ⋅TwS

This method of determining the relationships between workpiece frame and the two robotic base frames has two merits. One is that when one robot of the two robots cannot reach the selected three points, the two relationships can still be determined. The second advantage is that this method saves almost half the time used in previous workpiece frame calibration methods.

## 3. Trajectory Planning Approach for Inspecting Semi-Enclosed Workpieces in a DRNDT System

Nowadays, the offline trajectory planning method, which utilizes a 3D CAD model of a part to generate a tool path based on CAM software, is becoming more and more popular. It is more efficient and provides certain flexibility to the changes of products to testing [19].

Although it is expedient to generate a good raster scan trajectory for NDT probes as demonstrated by Section 2.1, if the trajectory path is generated by a customized software developed for 5-axis machine tool rather than for robot NDT system, post-processing is essential before applying the trajectory to the 6-axis robot NDT system [20]. One set of trajectory data generated for machine tool is [*x*, *y*, *z*, $t_{x}$, $t_{y}$, $t_{z}$], which is composed of position parameters *x*, *y*, *z* and unit normal vectors $t_{x}$, $t_{y}$, $t_{z}$.

According to the principle of conventional ultrasonic testing, the ultrasonic beam should be perpendicular to the surface of the workpiece in real time during the inspection. The principle of motion control of an industrial robot is to completely align the tool frames {TM}/{TS} with the auxiliary discrete point frame {$F_{i}$} (refer to Figure 4).

**Figure 6:**
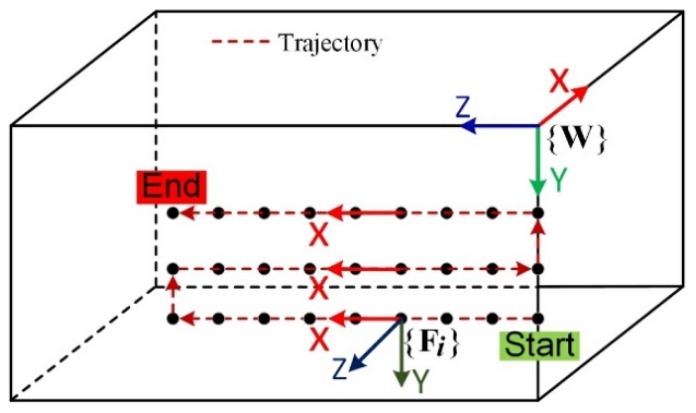
Auxiliary discrete point frame {$F_{i}$}.

To ensure the robots move in correct positions and orientations and do not collide with the workpiece, an algorithm is proposed to establish the auxiliary discrete frames for trajectory points ($P_{i}$), called the X-axis constraint method. In addition, in order to completely constrain the orientations of the robotic end effectors, it is an indispensable step to convert the normal vectors (vector direction cosines) of the discrete points to Euler angles relative to the frame {W} according to the X-Y-Z Euler angle principle, as shown in Figure 6.

**Figure 7:**
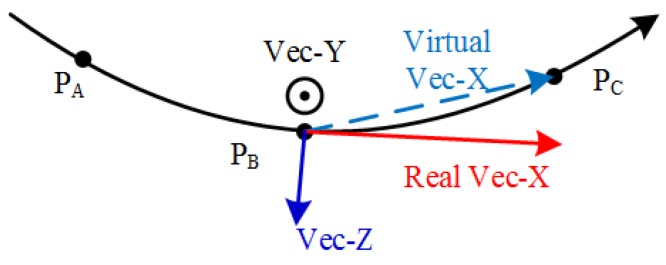
Correction process of X-axis of discrete point frame. Virtual Vec_X is the uncorrected X-axis of the frame. Real Vec_X is the corrected X-axis of the frame.

According to the X-axis constraint method, the X axes of all discrete points frames {$F_{i}$} are defined as the direction of the initial motion (point from the first point to the next point; the adjacent line points to the previous point from the current point) and tangent to the trajectory. Z-axis directions of discrete point frames take the directions of the outer normal vectors of the surface at the discrete points. The Y-axis is automatically obtained by the cross product of Z and X. The disadvantage of this method is that when the trajectory is not a straight line, the X-axis is not the tangential line of the trajectory and needs to be corrected. This is shown in Figure 7.

The specific method is as follows.

The X-axis of the initial discrete point frame is named virtual Vec_X denoted by $ox^{\prime }$:

(10) $$ox^{\prime }=\left(x_{c}-x_{b},\;y_{c}-y_{b},z_{c}-z_{b}\right).\;$$

The Z-axis of the discrete point frame is the tool axis vector, i.e.,

(11) $$oz=\left(t_{x},t_{y},t_{z}\right).$$

The Y-axis of the discrete point frame is the cross product of Z and $X^{\prime }$:

(12) $$oy=oz\times ox^{\prime }.$$

The real X-axis denoted by ox is corrected by the cross product of Y and Z, i.e.,

(13) ox=oy×oz.

The combination of these vectors constitutes the rotation matrix R of the discrete point frame {$F_{i}$} relative to the workpiece frame {W}.

(14) $$R=\left[\begin{array}{ccc} n_{x} & o_{x} & t_{x}\\ n_{y} & o_{y} & t_{y}\\ n_{z} & o_{z} & t_{z} \end{array}\right]$$

In Equation (14), the first column represents the projection of the X-axis of frame {$F_{i}$} in the workpiece frame, the second column represents the projection of the Y-axis of frame {$F_{i}$} in the workpiece frame, and the third column represents the projection of the Z-axis of frame {$F_{i}$} in the workpiece frame.

In order to ensure the smooth movement of the robots during the detection process and no risk of collision, all the X-axis directions of the discrete point frames are constrained according to the X-axis constraint method. In this way, it is possible to ensure that the robots inspect the entire workpiece with a slight change in orientation.

According to robotics, the rotation matrix $R_{XYZ}$ is expressed by Euler angles, as follows:

(15) $$\begin{array}{cc} R_{XYZ}= & R_{X(\alpha )}R_{Y(\beta )}R_{Z(\gamma )}=\left[\begin{array}{ccc} 1 & 0 & 0\\ 0 & c\alpha & -s\alpha \\ 0 & s\alpha & c\alpha \end{array}\right]\left[\begin{array}{ccc} c\beta & 0 & s\beta \\ 0 & 1 & 0\\ -s\beta & 0 & c\beta \end{array}\right]\left[\begin{array}{ccc} c\gamma & -s\gamma & 0\\ s\gamma & c\gamma & 0\\ 0 & 0 & 1 \end{array}\right]\\ = & \left[\begin{array}{ccc} c\beta c\gamma & -c\beta s\gamma & s\beta \\ s\alpha s\beta c\gamma +c\alpha s\gamma & -s\alpha s\beta s\gamma +c\alpha c\gamma & -s\alpha c\beta \\ -c\alpha s\beta c\gamma +s\alpha s\gamma & c\alpha s\beta s\gamma +s\alpha c\gamma & c\alpha c\beta \end{array}\right]=\left[\begin{array}{ccc} r_{11} & r_{12} & r_{13}\\ r_{21} & r_{22} & r_{23}\\ r_{31} & r_{32} & r_{33} \end{array}\right], \end{array}$$

where α, β, and γ are the Euler angles that represent the rotation angles of the discrete point frame relative to the workpiece frame in the order of X-Y-Z. cosα and sinα are abbreviated to cα and sα for clarity. α, β, γ has a unit of degree, i.e.,

(16) $$R_{XYZ}=\left[\begin{array}{ccc} r_{11} & r_{12} & r_{13}\\ r_{21} & r_{22} & r_{23}\\ r_{31} & r_{32} & r_{33} \end{array}\right]=R=\left[\begin{array}{ccc} n_{x} & o_{x} & t_{x}\\ n_{y} & o_{y} & t_{y}\\ n_{z} & o_{z} & t_{z} \end{array}\right].$$

By Equation (16), we have

(17) $$cos\beta =\pm \sqrt{r_{23}^{2}+r_{33}^{2}}=\pm \sqrt{t_{y}^{2}+t_{z}^{2}}.$$

In Equation (17), the positive value is used. In other words, the value range of β is set to (−90, 90), i.e.,

(18) $$cos\beta =\sqrt{r_{23}^{2}+r_{33}^{2}}=\sqrt{t_{y}^{2}+t_{z}^{2}}.$$

If cosβ≠0, we have

(19) $$\begin{array}{c} \alpha =Atan2\left(-r_{23}\;,\;r_{33}\right)\times 180/\pi \\ \beta =Atan2\left(r_{13}\;,\;\sqrt{r_{23}^{2}+r_{33}^{2}}\right)\times 180/\pi \;\\ \gamma =Atan2\left(-r_{21}\;,\;r_{11}\right)\times 180/\pi \end{array}.$$

If cosβ=0, the matrix $R_{XYZ}$ is reduced to

(20) $$R_{XYZ}=\left[\begin{array}{ccc} 0 & 0 & s\beta \\ s\left(\alpha +\gamma \right) & c\left(\alpha +\gamma \right) & 0\\ -c\left(\alpha +\gamma \right) & s\left(\alpha +\gamma \right) & 0 \end{array}\right].$$

Then, α is taken as 0, so we have

(21) $$\begin{array}{c} \alpha =0\\ \beta =Atan2\left(r_{13}\;,\;0\right)\times 180/\pi \\ \gamma =Atan2\left(r_{21}\;,\;r_{22}\right)\times 180/\pi \end{array}.$$

The trajectory data prepared from the CAM software is post-processed using Equation (10)–(21). The constrictions adopted in the algorithm allow the system to perform NDT of semi-enclosed workpieces smoothly.

## 4. Experiments Validation of the X-Axis Constraint Method

Experiments were performed on a rectangular semi-enclosed box and a cylindrical workpiece to verify the feasibility and correctness of the proposed trajectory processing method. Experiments were carried out on the DRNDT system, as shown in Figure 5. The master robot was STAUBLI Rx160 and the slave robot was STAUBLI Tx90xL. In addition, an extension arm was installed on the master robot.

### 4.1. Validation of the Rectangular Semi-Enclosed Box Trajectory

A trajectory was planned for one side of the rectangular semi-enclosed box shaped workpiece. The area of the trajectory was 100 mm × 200 mm. Its thickness was 12 mm. The movement mode was zig-zag. To improve the readability of the track image, the step pitch (between two lines) was set to 10 mm and the sampling pitch (between two discrete points) was set to 0.75 mm. 

**Figure 8:**
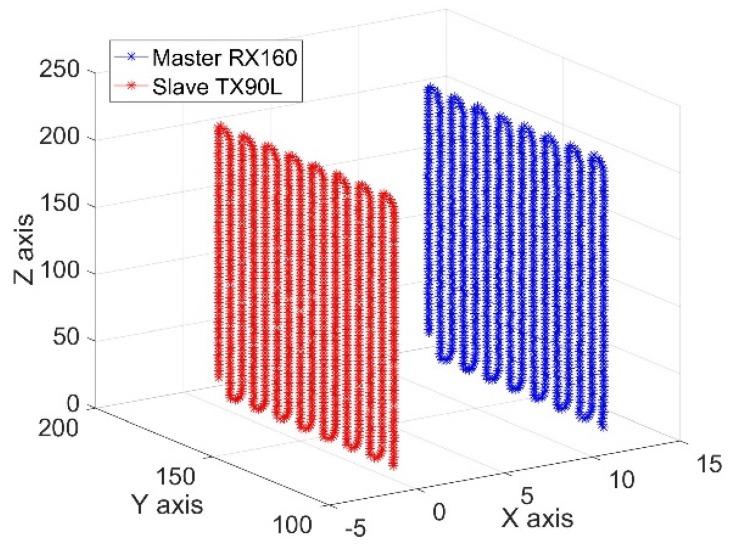
The trajectories of the robotic end effectors.

**Figure 9:**
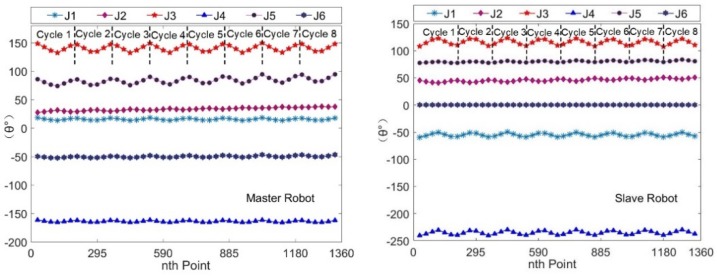
The curves of the joint angles of the two robots.

The Cartesian coordinates of the motion trajectory of the robotic end effectors and the corresponding robotic joints angles were collected for about 1360 points. The trajectory curves relative to frame {W} based on these data are shown in Figure 8, which shows that the trajectory curves of the two robots were parallel raster scanning lines. The joints angles curves of the two robots are shown in Figure 9. These smooth and continuous curves demonstrated that the two robots ran smoothly and no collisions occurred. The curves of J1, J3, J4, and J5 of the master robot and J1, J3, and J4 of the slave robot made a significant reciprocating motion, which corresponded exactly to the raster path. 

### 4.2. Validation of the Cylindrical Workpiece Trajectory

The X-axis constraint method is not only suitable for rectangular semi-enclosed box shaped workpieces, but also for other similar semi-enclosed workpieces. To further verify the correctness of the method, a cylindrical semi-enclosed workpiece was used for the test object. This cylindrical workpiece had an outer diameter of 250 mm. The thickness was 25 mm. The step pitch (between two lines) was set to 15 mm and the sampling pitch was set to 2 mm.

**Figure 10:**
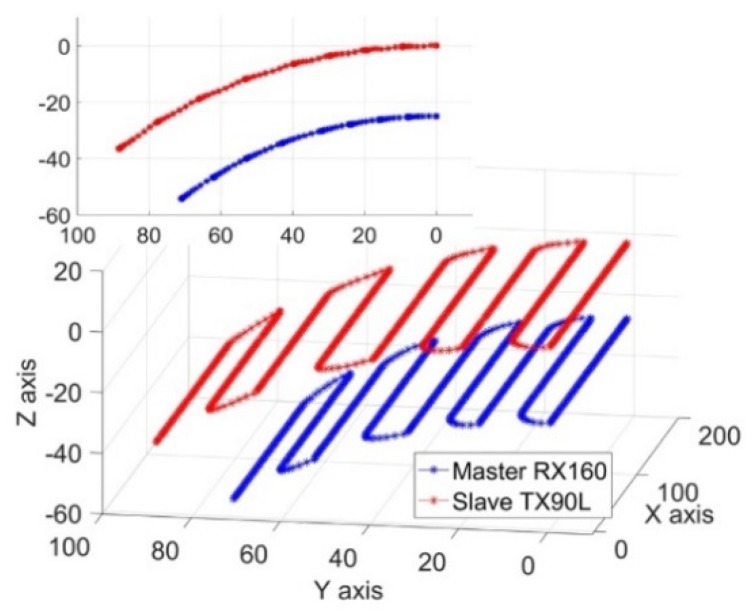
The trajectories of the robotic end effectors.

**Figure 11:**
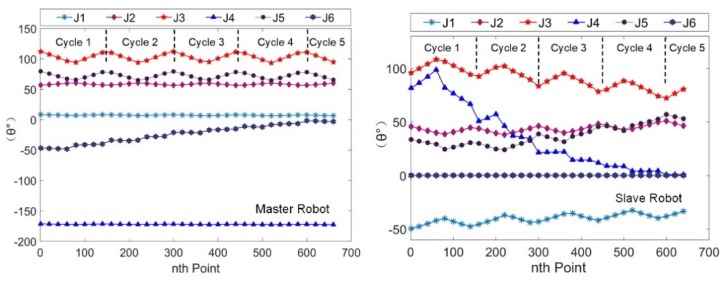
The curves of joint angles of the two robots.

Due to a smaller detection area and a larger sampling pitch, 661 sets of Cartesian coordinate data and joint angle data were collected. The Cartesian trajectory curves of the two robotic end effectors relative to frame {W} are shown in Figure 10. The curves revealed that the trajectories of the robotic tools were parallel raster scan lines. The joint angle curves of the two robots are shown in Figure 11. The results were very similar to that of the rectangular semi-enclosed box shaped workpiece. Such a constraint ensured that the extension arm was substantially parallel to the rotary axis of the cylindrical workpiece.

The scanning trajectory verification experiments of the two workpieces basically illustrated the practicability of the dual-robot NDT scheme with one robot clamping an extension arm for semi-enclosed workpiece non-destructive testing and the correctness of the X-axis constraint trajectory planning method for inspecting semi-enclosed workpieces in a DRNDT system. Ultrasonic NDT was then performed on the two workpieces.

## 5. Ultrasonic NDT Experiments of the Dual-Robot NDT System

### 5.1. Sound Field Analysis of the Probe Used in the Experiment

Ultrasonic testing ensures that the ultrasonic energy introduced into the workpiece is relatively maximized. In order to accurately control the distance of the ultrasonic probe from the surface of the workpiece (that is, the water path distance), the sound field of the 2.25 MHz ultrasonic probe (element size 12.7 mm) in water was calculated based on the multi-Gaussian beam model. The multi-Gaussian beam model describes the sound field distribution of the piston probe with a simple analytical expression. Its basic idea is to superimpose several single Gaussian sound beams [21]. The beam pressure distribution for a piston probe in liquid based on the multi-Gaussian beam model can be written as

(22) $$\frac{p\left(x,y,z,\omega \right)}{\rho_{1}c_{p}v_{0}\left(\omega \right)}=\sum_{i=1}^{15}\frac{A_{n}}{1+iB_{n}z/D_{r}}\exp\left(ik_{p}D_{r}\right)\exp\left(i\omega \left(\frac{1}{2}X^{T}{\left(M_{1}^{p}\left(z\right)\right)}_{n}X\right)\right),$$

where:

$v_{0}\left(\omega \right)$ is the sound speed radiated by the piezoelectric crystal piece at z = 0;
$\rho_{1}$ is the medium density ($\mathrm{kg}/m^{3}$);
$c_{p}$ is sound speed (m/s);
z is the direction of sound propagation (mm);
$k_{p}$ is the wave numbers;
$D_{r}=\frac{k_{p}a^{2}}{2}$ is the Rayleigh distance;
a is the radius of the transducer (m);
$X={\left[x,y\right]}^{T}$;
$A_{n},B_{n}$ are the complex Gaussian coefficient;
${\left[M_{1}^{P}\left(z\right)\right]}_{n}=\left[\begin{array}{cc} \frac{iB_{n}/c_{p}D_{r}}{1+iB_{n}x_{3}/D_{r}} & 0\\ 0 & \frac{iB_{n}/c_{p}D_{r}}{1+iB_{n}x_{3}/D_{r}} \end{array}\right]$.

**Figure 12:**
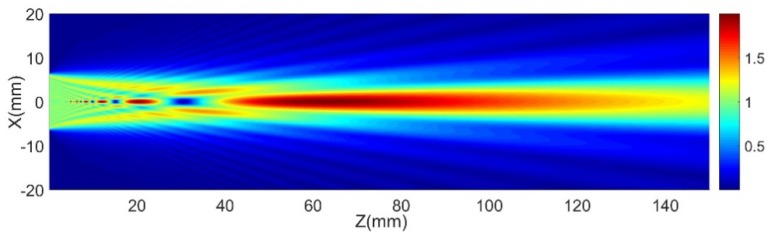
The sound field of the 2.25 MHz probe.

The sound field of the probe used in the experiments is shown in Figure 12. It showed that the optimal water path distance of the selected probe was about 58–70 mm.

### 5.2. Ultrasonic Testing of the Rectangular Semi-Enclosed Box Shaped Workpiece

Generally, the DRNDT system utilized the water-jet-coupling ultrasonic transmission testing method. Ultrasonic equipment in the DRNDT system mainly included a couple of 2.25 MHz ultrasonic probes (element size 12.7 mm, damping 200 Ohm, OLYMPUS unfocused probes), a pulser-receiver (brand and model: OLYMPUS 5077PR), and data acquisition board (brand and model: Acquisition Logic AL12250).

**Figure 13:**
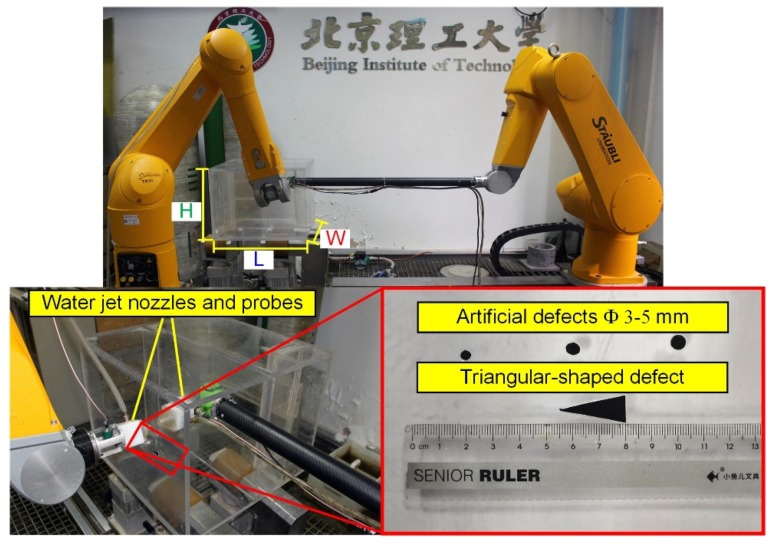
DRNDT system and the inspection of the rectangular, semi-enclosed box-shaped workpiece. L = 520 mm, W = 500 mm, H = 400 mm.

The workpiece under testing was the one mentioned in Section 4.1. Its material was polymethyl methacrylate (PMMA), which is a colorless, transparent material. The length (L), width (W), and height (H) of the rectangular semi-enclosed workpiece were 520 mm, 500 mm, and 400 mm, respectively, and the thickness was 12 mm. Three circular-shaped defects (Φ3 mm, Φ4 mm, and Φ5 mm) and one triangular-shaped defect (the bottom length was 10 mm and the height was 25 mm) were pasted on the surface of the workpiece, as shown in Figure 13. A raster scan was performed and the step pitch and sampling pitch were set to 0.5 mm. 

**Figure 14:**
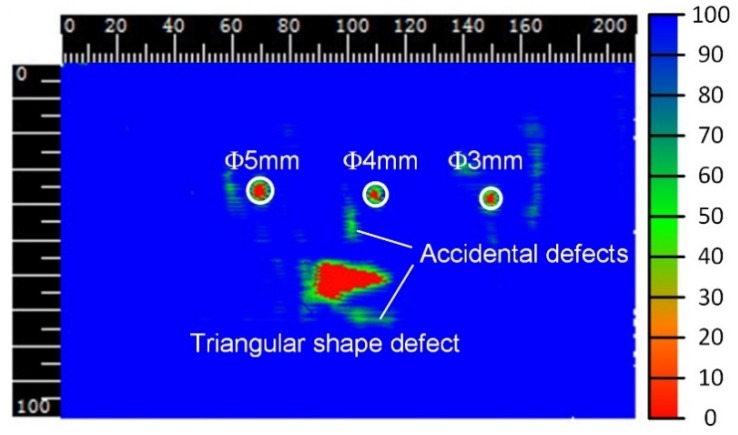
C-scan image of the testing area of the rectangular semi-enclosed box.

A rectangular area 200 mm × 100 mm was scanned and 78,658 sets of position data and the corresponding ultrasonic data were collected. The C-scan image based on these data is shown in Figure 14. The color from blue to red indicates the ultrasonic A-scan signal from strong to weak, that is, red represents a defect.

From the C-scan images, all the artificial defects from 3 mm to 5 mm and the triangular-shaped defect on the workpiece were clearly detected; the sizes of the defects and the positions can be directly acquired from the C-scan image. In addition, some irregular-shaped defects also emerged on the C-scan image. Since the workpiece was transparent, visual inspection revealed that there were no defects inside the workpiece and on the surface of the workpiece before pasting the artificial defects, so it can be judged that these accidental defects were caused by bubbles and small wrinkles between the tape and the workpiece. The C-scan image also has a very good ability to restore the triangular defects.

### 5.3. Ultrasonic Testing of the Cylindrical Semi-Enclosed Workpiece

**Figure 15:**
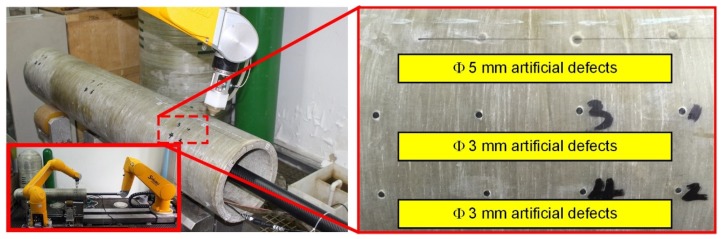
DRNDT system and the inspection of cylindrical workpiece.

In order to fully verify the flexibility of the DRNDT system, the semi-enclosed workpiece with a curved surface in Section 4.2, of which the outer diameter was 250 mm, the thickness was 25 mm, and the length was 1500 mm, was used as the workpiece to be inspected. Its material was epoxy resin-based glass fiber. It was made by winding glass fibers pre-impregnated with epoxy resin. Some artificial defects were made in the workpiece, as shown in Figure 15. There were three rows of hole-shaped defects and the defects’ sizes of each row were the same. The hole diameters were 3 mm, 3 mm, and 5 mm. All holes had the same depth of 10 mm.

**Figure 16:**
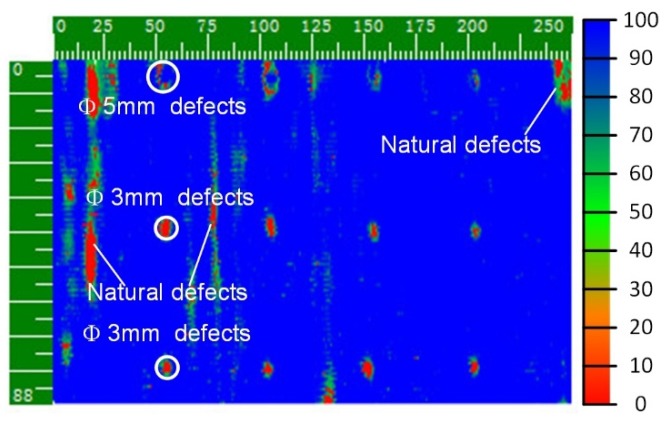
C-scan result of the testing area of the cylindrical workpiece.

The step pitch and sampling pitch were set to 0.75 mm and 43,649 sets of position data and the corresponding ultrasonic data were collected. The C-scan image based on these data is shown in Figure 16.

Figure 16 clearly shows the sizes and positions of all artificial defects and some irregular-shaped natural defects located in the workpiece body. Careful observation shows that the centers of the 5 mm artificial defects were not highly discriminating. This is because the material that filled in the hole-shaped artificial defects sank into the bottoms of the holes and acted as a coupling medium, losing the essence of a defect. In addition, the detection results of the two rows of 3 mm artificial defects showed that the system had the same detection resolution for defects in different positions of space.

**Figure 17:**
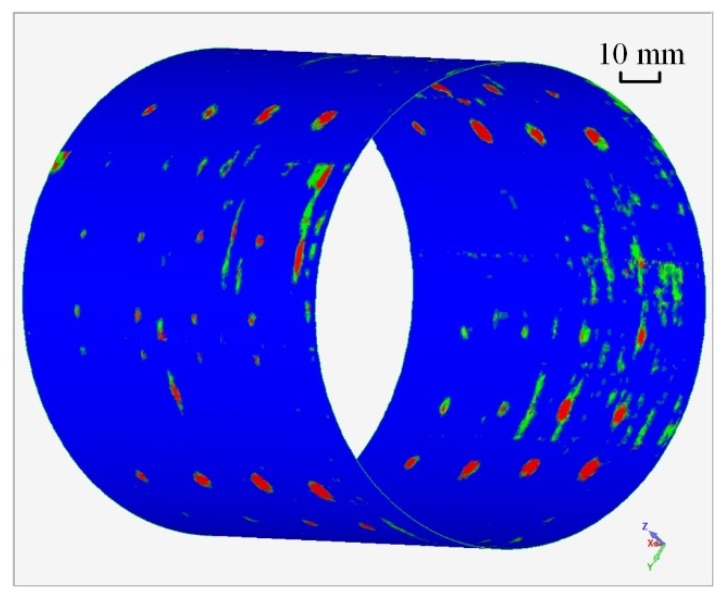
3D C-scan result of the cylindrical workpiece.

In total, 349,211 sets of position data and the corresponding ultrasonic data were collected for a section of the cylinder (250 mm along the axis). The 3D C-scan imaging based on these data is shown in Figure 17. The image is displayed in 3D for more intuitive and vivid results.

## 6. Conclusions

To cope with the NDT challenge of semi-enclosed workpieces, a dual-robot-based ultrasonic NDT system scheme was proposed. The coordinate frames and coordinate transformation method related to the cooperative motion control of the dual-robot system were discussed. A trajectory planning approach for inspecting semi-enclosed workpieces in DRNDT system was studied and an X-axis constraint method was proposed to process the trajectory data generated by CAM software. An auxiliary coordinate frame was established at each discrete trajectory point according to this method to minimize the orientation change of the robotic tools during the whole detection process.

The trajectory validation experiments were implemented so that the two robots moved smoothly and no collisions occurred when the robots moved in the trajectory. This was processed by the X-axis constraint method.

Ultrasonic C-scan testing experiments utilizing the optimal water path distance, about 58–70 mm, determined by simulation result of a multi-Gaussian beam model, were carried out on a rectangular semi-enclosed box-shaped workpiece and on a cylindrical workpiece. All the obtained C-scan images clearly presented not only the artificial defects, but also the natural defects. Further, for the rectangular semi-enclosed box, due to the bubbles and small wrinkles between the tape and the workpiece, some irregular-shaped defects also emerged on the C-scan image. In addition, the clear red triangle on the C-scan image illustrated that the DRNDT system had a very good ability to restore the triangular defects. For the cylindrical workpiece, since the material that filled in the hole-shaped artificial defects sank into the bottoms of the holes, the performance of the artificial defects was invalid. Therefore, the centers of the 5 mm artificial defects in the C-scan image were not highly discriminating. The detection results of the two rows of 3 mm artificial defects showed that the system had the same detection resolution for defects in different positions of space. Finally, the 3D C-scan images made the testing results clearer and more vivid.

According to the above analysis, a dual-robot NDT system with one robot equipping an extension arm is suitable for the NDT of semi-enclosed workpieces.
